# Randomized Comparison of Mobile and Web-Tools to Provide Dementia Risk Reduction Education: Use, Engagement and Participant Satisfaction

**DOI:** 10.2196/mental.3654

**Published:** 2014-12-22

**Authors:** Elodie O'Connor, Maree Farrow, Chris Hatherly

**Affiliations:** ^1^ Alzheimer's Australia Parkville Australia; ^2^ Alzheimer's Australia Parkville Australia; ^3^ Centre for Research on Ageing, Health and Wellbeing The Australian National University Canberra Australia; ^4^ Alzheimer's Australia Scullin Australia

**Keywords:** dementia, Alzheimer, engagement, health communication, Internet, intervention, mobile phone, risk reduction behavior, user perceptions, mhealth

## Abstract

**Background:**

Encouraging middle-aged adults to maintain their physical and cognitive health may have a significant impact on reducing the prevalence of dementia in the future. Mobile phone apps and interactive websites may be one effective way to target this age group. However, to date there has been little research investigating the user experience of dementia risk reduction tools delivered in this way.

**Objective:**

The aim of this study was to explore participant engagement and evaluations of three different targeted smartphone and Web-based dementia risk reduction tools following a four-week intervention.

**Methods:**

Participants completed a Web-based screening questionnaire to collect eligibility information. Eligible participants were asked to complete a Web-based baseline questionnaire and were then randomly assigned to use one of the three dementia risk reduction tools for a period of four weeks: (1) a mobile phone application; (2) an information-based website; and (3) an interactive website. User evaluations were obtained via a Web-based follow-up questionnaire after completion of the intervention.

**Results:**

Of 415 eligible participants, 370 (89.16%) completed the baseline questionnaire and were assigned to an intervention group; 200 (54.05%) completed the post-intervention questionnaire. The average age of participants was 52 years, and 149 (75%) were female. Findings indicated that participants from all three intervention groups reported a generally positive impression of the tools across a range of domains. Participants using the information-based website reported higher ratings of their overall impression of the tool, F_2,191_=4.12, *P*=.02; how interesting the information was, F_2,189_=3.53, *P*=.03; how helpful the information was, F_2,192_=4.15, *P*=.02; and how much they learned, F_2,188_=3.86, *P*=.02. Group differences were significant between the mobile phone app and information-based website users, but not between the interactive website users and the other two groups. Additionally, participants using the information-based website reported significantly higher scores on their ratings of the ease of navigation, F_2,190_=4.20, *P*=.02, than those using the mobile phone app and the interactive website. There were no significant differences between groups on ratings of ease of understanding the information, F_2,188_=0.27, *P*=.76. Most participants from each of the three intervention groups indicated that they intended to keep using the dementia risk reduction eHealth tool.

**Conclusions:**

Overall, results indicated that while participants across all three intervention groups reported a generally positive experience with the targeted dementia risk reduction tools, participants using the information-based website provided a more favorable evaluation across a range of areas than participants using the mobile phone app. Further research is required to investigate whether targeted dementia risk reduction tools, in the form of interactive websites and mobile apps, can be improved to provide benefits above those gained by providing static information alone.

## Introduction

### Background

With increasing life expectancy, the global burden of dementia is rapidly increasing, with numbers expected to almost double every 20 years from the 35.6 million people affected in 2010 [1]. Currently, no effective treatments exist to stop or reverse progression of dementia. However, several modifiable health and lifestyle factors have consistently been found to be associated with the risk of developing dementia [2-5]. Factors that may increase the risk of dementia include high blood pressure, midlife high total cholesterol, diabetes, midlife obesity, and smoking; while factors that may decrease the risk include regular physical exercise, mental and social activity, and the Mediterranean diet [2-5]. There is compelling evidence that managing vascular risk factors and remaining mentally and physically active from midlife may reduce the risk or delay the onset of dementia or cognitive decline in late life for individuals, and reduce the future incidence in the population [5-8].

A significant international research effort is currently aimed at developing and evaluating targeted dementia risk reduction interventions. Because the prevalence of dementia increases exponentially with age (from approximately 1-2% at age 65 to 20% at age 85 [1]), most of this research focuses on older people. However, as the underlying pathology and resulting brain damage precede the symptoms of dementia by years or decades [9] and many risk and protective factors have the strongest effect in midlife [10], developing late life interventions is only one part of the required preventative health approach.

While the direct impact of late life interventions on dementia incidence can be assessed by clinical trials conducted over a few years, this is impractical for midlife interventions due to the long interval before the outcome of interest (dementia diagnosis) could be assessed. However, existing epidemiological evidence suggests it is likely that encouraging people to change their behavior and maintain their physical and cognitive health in midlife can have a significant impact on reducing the prevalence of dementia in the future [6,8]. Despite this potential, a large number of Australian adults have limited knowledge about dementia risk factors [11,12].

### eHealth Tools

Over 80% of Australians are Internet users [13]. eHealth interventions (health care using the Internet; eg, websites and mobile phone apps) are increasingly being utilized to promote health behavior change [14,15]. eHealth interventions are advantageous because they offer convenience and anonymity to the user [16-18], they allow for individualized, tailored feedback [15], and they have the potential to reach large audiences at a low cost [19,20].

eHealth interventions have been reported to be an effective method for increasing knowledge and/or enabling healthy behavior change [21]; for example, in areas such as physical activity [22], and overweight and obesity prevention [23]. This suggests that eHealth interventions are efficacious for the promotion of healthy lifestyles. However, to date there has been limited high quality research evaluating eHealth intervention programs designed to target multiple behaviors [24,25].

### Alzheimer’s Australia’s Dementia Risk Reduction Program

Alzheimer’s Australia (Australia’s national dementia association) developed a community education program designed to inform people about what they can do to reduce their risk of dementia (Your Brain Matters). It is based on the scientific evidence and focuses on ten health and lifestyle behaviors that have been identified as modifiable risk and protective factors: alcohol use, blood pressure, body weight, cholesterol, diabetes, diet, mental activity, physical activity, smoking, and social activity. The program initially focused on community education forums and printed resources, but now includes eHealth tools such as a website and mobile phone app. In designing the eHealth tools, the aim was to disseminate the current evidence for modifiable risk and protective factors associated with dementia risk to the Australian community using accessible and engaging modalities. To aid development of these eHealth tools, Alzheimer’s Australia reviewed the relevant literature and sought expert advice on the recommendations being made. Alzheimer’s Australia staff and consumer advisors provided feedback about the appropriateness of the content for the general public.

The original website developed for this program was evaluated in a previous study [25]. Results indicated that while participants found the website to be interesting, informative, and helpful, additional personalized and interactive resources were desired. Resources to assess and address individual risk factors were rated as potentially very useful [25]. Further resources were developed with the aim of enhancing the website and providing resources on a mobile platform in order to better assist people to implement behavior change, rather than providing static information alone. These personalized and interactive resources include: a brain health survey with results indicating how brain healthy users’ current lifestyles are, tailored activity suggestions, tools for recording weekly goals and activities, and brief progress surveys for each health behavior.

While evaluation of the effectiveness of the dementia risk reduction eHealth tools will require assessment of outcomes such as improved knowledge and behavior change, an important first step is evaluating whether the tools are acceptable to intended users. An evaluation of user experiences and perceptions of these eHealth tools was therefore the focus of the present study. The Alzheimer’s Australia dementia risk reduction resources are continually being updated and revised, and an important aspect of these revisions is to ensure they remain relevant and useful for the user, while being easy to access and navigate. Thus, an evaluation which focuses on the user experience of each of these resources is essential.

### Current Study

eHealth tools may present a feasible method for providing dementia risk reduction resources to the Australian community, particularly to those in midlife. However, there has been limited research, to date, examining the user experience. The aim of the present study was to explore participant preferences regarding three eHealth interventions. It was hypothesized that participants would provide a more positive evaluation of the interactive eHealth tools (an interactive mobile phone app or an interactive Web-based program) than for a static information-only website.

## Methods

### Participants

**Figure 1:**
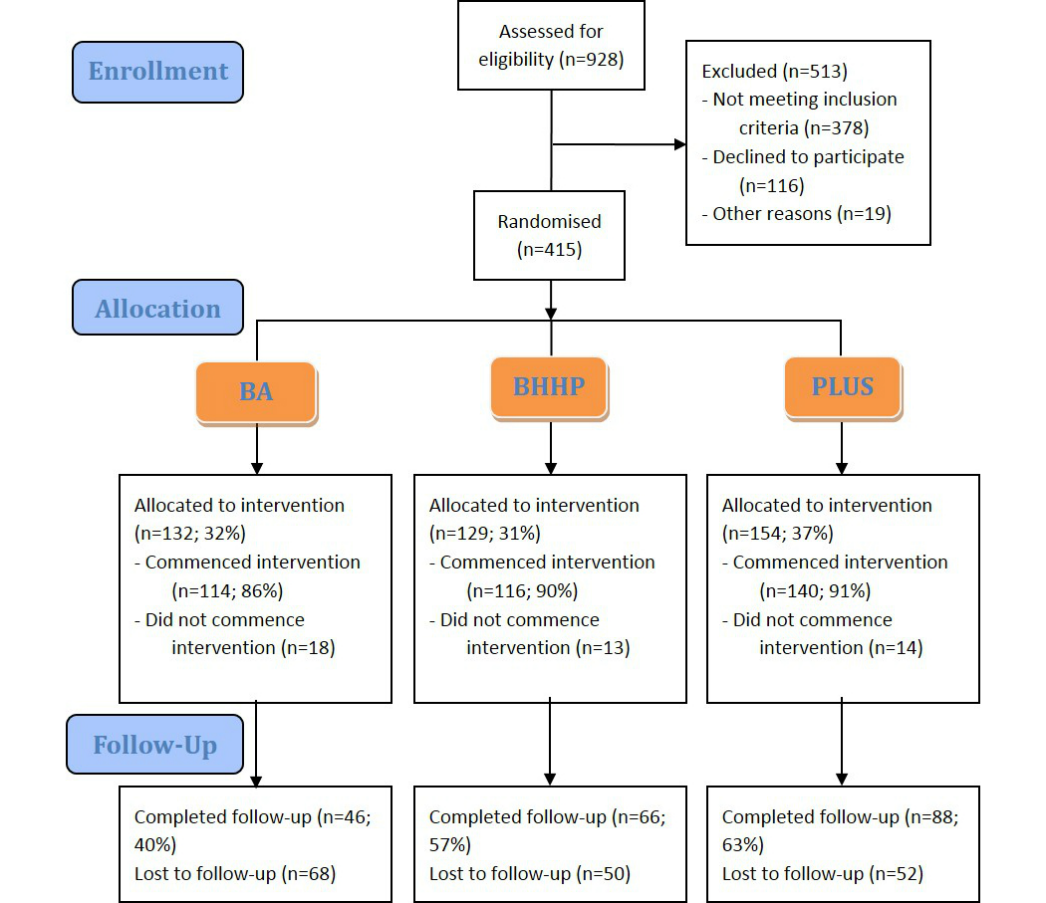
Flow diagram detailing the involvement of participants in the study. BA: BrainyApp (mobile phone/tablet app); BHHP: Brain-Heart Health Program (information only website); PLUS: Brain-Heart Health Plus Program (interactive Web-based program).

Participants were recruited through a range of targeted online and media promotions, including newspaper advertisements, radio interviews, online forum advertising and social media posts. A total of 928 people provided online consent to participate in the research project and completed a screening questionnaire. Of these, 135 (14.6%) had invalid screening tools (eg, withdrew prior to completing the screen or had duplicate entries). Of the 793 valid screens, 415 (44.7%) were eligible. The primary reason for ineligibility was not having an Apple device (n=346, 91.5%; an Apple device being required to use the mobile phone app/tablet tool). Additional eligibility criteria included being 18 years of age or older, fluent in English, healthy enough to undertake physical exercise, and not having a psychiatric or neurological condition. Figure 1 details a flow diagram for participants.

Three hundred and seventy (89.16%) eligible participants completed the online baseline questionnaire and thus entered the study. Of these, 200 (54.05%) continued through to completion of the online four-week post-intervention questionnaire, with the remaining 170 not responding to follow-up reminder emails.

### Procedure

In this study, participants made contact initially through an advertisement which provided a link to an online screening tool. Eligible participants were then randomly allocated to use BrainyApp (an app available on Apple devices; BA group), or the Brain-Heart Health Program (an information-based website; BHHP group), or the Brain-Heart Health Plus Program (an interactive website; PLUS group) and invited, via email, to complete a baseline questionnaire and then engage with the app, information-based website, or interactive website for a period of four weeks. During this time, participants’ use of the eHealth tool was monitored by automatic logging of the frequency and duration of their use of the app or website. A reminder email was sent halfway through the four week intervention, encouraging participants to continue using the eHealth tool. At the conclusion of the intervention period, participants were asked to complete a post-intervention questionnaire online. All participants were offered a $20 Woolworths (major supermarket chain) voucher as compensation for their time and effort. Ethical approval was obtained from the Australian National University Human Research Ethics Committee.

### Interventions

**Figure 2:**
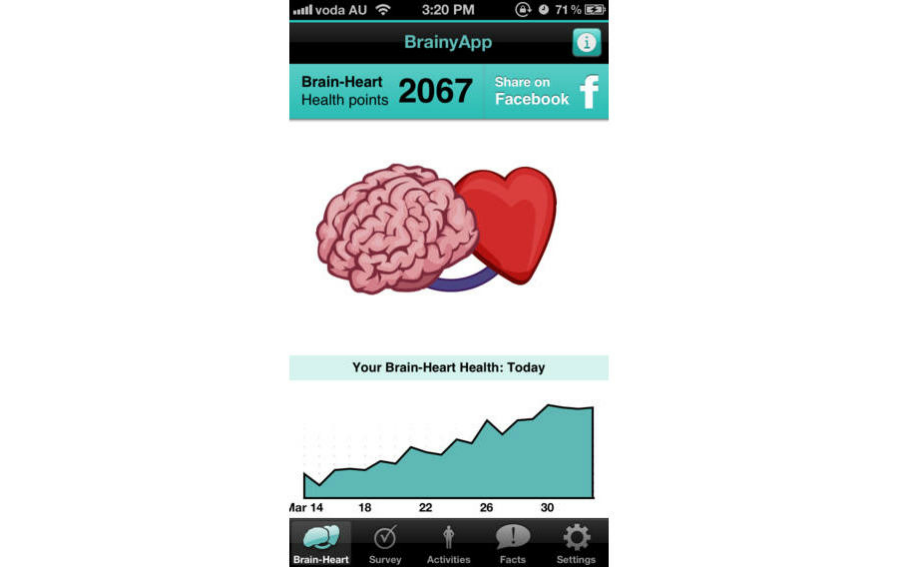
Screenshot of the BrainyApp “Brain-Heart Health points” page.

**Figure 3:**
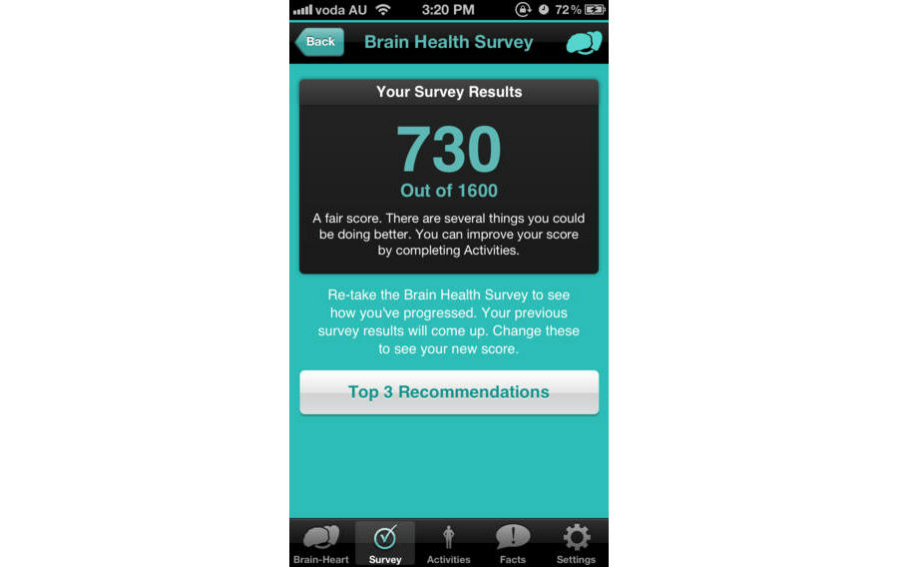
Screenshot of the BrainyApp “Brain Health Survey: Your Results” page.

BrainyApp is a mobile device application for iPhone, iPad, and iPod Touch. Figures 2 and 3 illustrate two screenshots from BrainyApp. This tool allows users to complete a brain health survey, which asks questions about current physical, social and mental activity, cardiovascular health, diet, smoking and drinking habits. The brain-heart health score achieved indicates how brain healthy the users’ current lifestyle is and particular areas for improvement are highlighted. Users can then engage in activities to improve in areas that may be increasing their dementia risk. If users record sufficient activities according to recommendations for dementia risk reduction, their brain-heart health score improves over time. Users can also read and share facts about dementia, the brain and how to keep their brain healthy. BrainyApp is publicly available (and has been downloaded over 300,000 times since its release in 2011), but participants in this study used a research version of the app that allowed monitoring of their usage.

**Figure 4:**
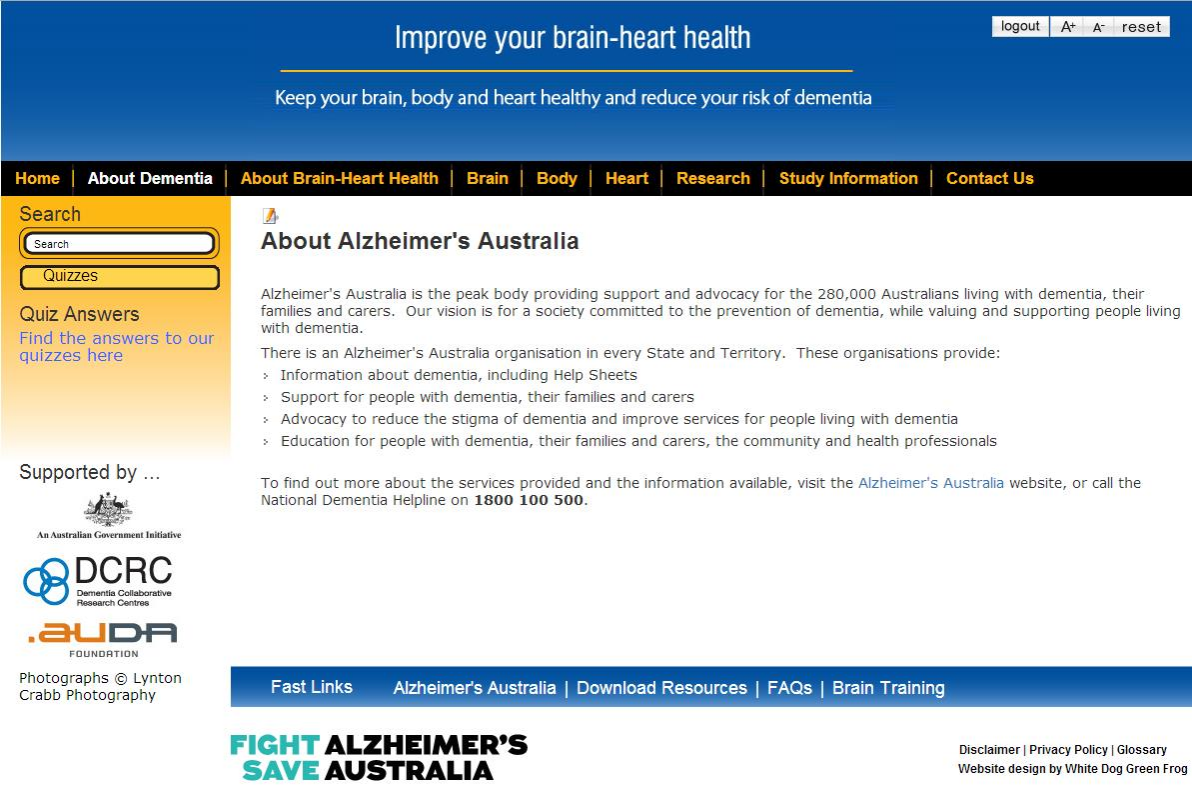
Screenshot of the Brain-Heart Health Program “About Alzheimer’s Australia” page.

**Figure 5:**
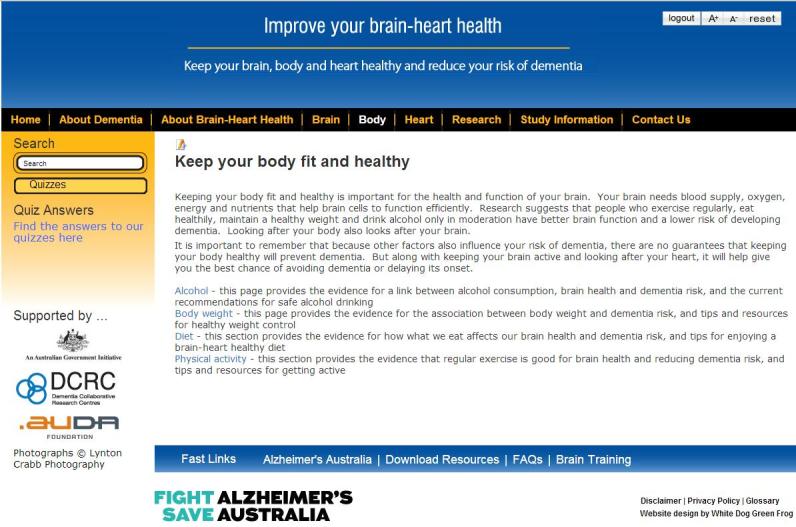
Screenshot of the Brain-Heart Health Program “Keep your body fit and healthy” page.

The Brain-Heart Health Program is an information-based website. Figures 4 and 5 illustrate two screenshots from the information-based website. This site provides static information only, and the information about risk and protective factors is presented in three sections–Brain, Body, and Heart. These sections explain the current evidence and provide some practical advice on how users can be brain healthy and reduce their risk of dementia, with links to additional relevant resources. Users can also learn about dementia, the brain, and how to keep their brain healthy. The information-based website was created specifically for this study, and was only accessible to participants with a log-in account.

**Figure 6:**
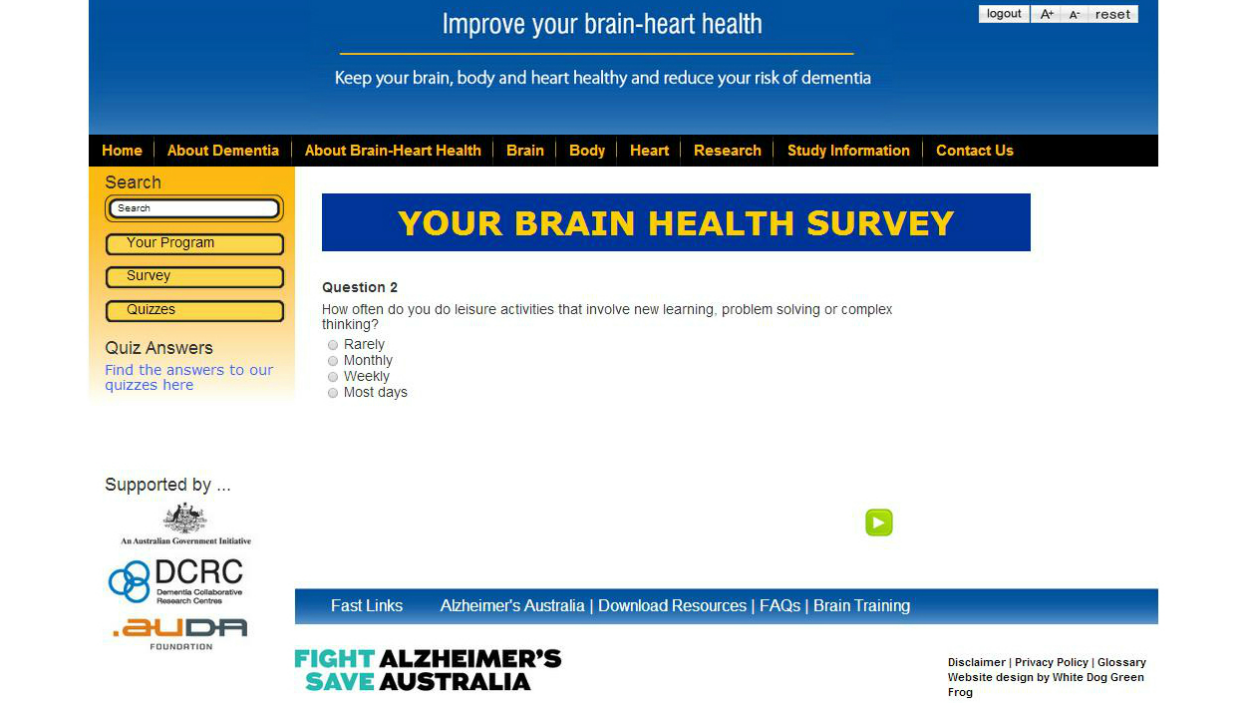
Screenshot of the Brain-Heart Health Plus Program “Your Brain Health Survey” page.

**Figure 7:**
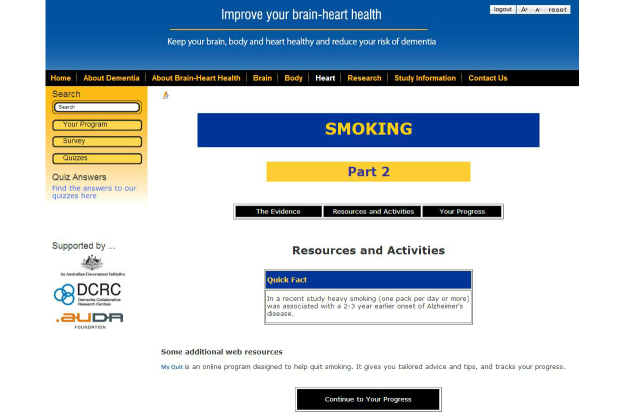
Screenshot of the Brain-Heart Health Plus Program “Smoking: Part 2” page.

The Brain-Heart Health Plus Program is an interactive Web-based brain health program. Figures 6 and 7 illustrate two screenshots from the interactive website. The information about risk and protective factors is presented in three sections–Brain, Body, and Heart. The interactive website also allows users to complete a brain health survey, which asks questions about current physical, social and mental activity, cardiovascular health, diet, smoking and drinking habits. Survey results indicate how brain healthy users’ current lifestyle is. Users are then provided with information about which areas could be improved to boost their brain health, and are given the opportunity to engage in recommended activities to improve in these areas. They are provided with links to additional relevant resources, research snapshots, planners for recording weekly goals and activities, and brief progress surveys. Additional health information, practical tips, and resources, were emailed to users halfway through the intervention. Users can also learn about dementia, the brain, and how to keep their brain healthy. The interactive website was created specifically for this study, and was only accessible to participants with a log-in account.

### Measures

#### Demographic Information

Participants were asked about their age, gender, country of birth, current living situation, and highest level of education. They were also asked about their current employment status, current occupation, whether they had ever worked as a health professional, and their annual household income (before tax). Finally, they were asked how often they download and use a new general app or health-related app on their Apple device, and how often they use the Internet to search for general and health-related information, on a five-point scale (from 1=never to 5=daily).

#### Evaluation Information

##### Overview

Participants were asked a series of questions about their use and evaluation of the tools during the four-week intervention. Questions were tailored specifically to each of the three intervention tools.

##### Use of eHealth Tools

Participants were asked whether they had heard of or used Alzheimer’s Australia’s Your Brain Matters or BrainyApp prior to the study, how they accessed the current intervention tool, how often they used it, how long they used it for each time, whether they were ever unable to access it, and whether they intended to keep using it. For the BA and PLUS groups, they were also asked whether they were surprised by their scores on the survey. Participants’ use of the eHealth tool was tracked throughout the four-week intervention; data included frequency and duration of use.

##### Evaluation of Intervention

Using a five-point scale, participants were asked about their overall impression of the intervention (from 1=terrible to 5=excellent), whether the information provided was interesting (from 1=not at all interesting to 5=very interesting), and easy to understand (from 1=very complex to 5=very simplistic), whether the intervention was easy to navigate (from 1=very difficult to 5=very easy), how helpful they found the intervention (from 1=not at all helpful to 5=very helpful), and how much they felt they learned (from 1=nothing at all to 5=a great deal).

### Analysis

IBM SPSS Statistics Version 21 was used to conduct the analyses. The average rate of missing data across the variables was low (no more than 3.0%); as a result, pairwise deletion was utilized for all analyses. A threshold of *P*<.05 was used for reporting statistical significance.

The analyses are presented in four parts. First, significant differences between completers and non-completers were explored. Next, the sample characteristics were described, including levels of eHealth usage prior to the study. Third, usability and access data were examined across the intervention groups, including both self-report data and user tracking. Finally, differences in evaluation scores between the intervention groups were examined using a series of one-way between-groups ANOVAs.

With power set at .80 and alpha=.05, the required sample size for each group in order to observe a medium effect was 52 [26]. Thus, the sample size for BHHP and PLUS groups was adequate, while the sample size for the BA group was limited.

## Results

### Comparison of Completers and Non-Completers

A series of independent *t* tests and chi-square tests for independence indicated the following significant differences between the 200 completers of the four-week follow-up and the 170 non-completers: education level, *χ* ^2^_5_=17.2, n=370, *P*=.004, Cramer’s *V*=.22, (95% CI 0.07-0.30); and employment status, *χ* ^2^_6_=12.9, n=369, *P*=.045, Cramer’s *V*=.19, (95% CI 0-0.26). Those who completed the follow-up were more likely to have an undergraduate degree (n=64, 32.0% vs n=32, 18.8%) and be retired or looking for work (n=59, 29.5% vs n=25, 14.8%), while those who did not complete the follow-up were more likely to have a postgraduate degree (n=65, 38.2% vs n=56, 28.0%) and working full-time (n=69, 40.8% vs n=63, 31.5%).

There were no significant differences found between completers and non-completers for age, *t*_368_=1.59, *P*=.11, mean difference=2.53 years, (95% CI -0.60 to 5.67); gender, *χ* ^2^_2_=2.8, n=366, *P*=.24, Cramer’s *V*=.09, (95% CI 0-0.18); country of birth, *χ* ^2^_1_=0.02, n=369, *P*=.99, phi=-.01, (95% CI 0-0.04); living situation, *χ* ^2^_3_=5.7, n=368, *P*=.13, Cramer’s *V*=.13, (95% CI 0-0.21); occupation, *χ* ^2^_12_=10.9, n=364, *P*=.53, Cramer’s *V*=.17, (95% CI 0-0.19); work as a health professional, *χ* ^2^_1_=0.9, n=369, *P*=.27, phi=-.06, (95% CI 0-0.16); or household income, *χ* ^2^_3_=4.1, n=360, *P*=.25, Cramer’s *V*=.11, (95% CI 0-0.19).

### Demographic Characteristics

Demographic characteristics of the participants are detailed in Table 1. Three-quarters of participants were female, with an average age of 52 years. Most participants were born in Australia, and lived with their partner and/or children. More than half of all participants had an undergraduate or postgraduate degree; the majority were employed either full-time or part-time. A wide range of occupations were represented; 19% (38 of 200) had worked as a health professional; and the vast majority had a household income of more than AUD$52,000 per annum.

A series of one-way between-groups ANOVAs and chi-square tests for independence indicated that there were no significant differences between intervention groups for the demographic characteristics: age, *F*_*2,197*_=0.28, *P*=.78; gender, *χ* ^2^_4_=7.9, n=198, *P*=.10, Cramer’s *V*=.14, (95% CI 0-0.22); country of birth; *χ* ^2^_2_=1.8, n=200, *P*=.41, Cramer’s *V*=.10, (95% CI 0-0.22); current living situation, *χ* ^2^_6_=3.8, n=199, *P*=.71, Cramer’s *V*=.10, (95% CI 0-0.14); education, *χ* ^2^_10_=8.2, n=200, *P*=.61, Cramer’s *V*=.14, (95% CI 0-0.17); employment status, *χ* ^2^_12_=9.6, n=200, *P*=.65, Cramer’s *V*=.16, (95% CI 0-0.17); occupation, *χ* ^2^­_24_=21.1, n=198, *P*=.63, Cramer’s *V*=.23, (95% CI 0-0.29); work as a health professional, *χ* ^2^_2_=1.8, n=200, *P*=.41, Cramer’s *V*=.10, (95% CI 0-0.22); or income, *χ* ^2^_6_=5.1, n=193, *P*=.53, Cramer’s *V*=.12, (95% CI 0-0.17).

**Table 1:** Demographic characteristics for all participants who completed the four-week follow-up.

Characteristics		BA (n=46)	BHHP (n=66)	PLUS (n=88)
		Mean (SD) or n (%)	Mean (SD) or n (%)	Mean (SD) or n (%)
Age, mean (SD)		52.26 (15.81)	53.18 (13.75)	51.35 (15.48)
**Gender,** n (%)				
	Male	16 (34.80)	9 (14.10)	23 (26.10)
	Female	30 (65.20)	55 (85.90)	64 (72.70)
	Other	0 (0.00)	0 (0.00)	1 (1.10)
**Country of birth,** n (%)				
	Australia	36 (78.30)	45 (68.20)	67 (76.10)
	Other	10 (21.70)	21 (31.80)	21 (23.90)
**Living situation,** n (%)				
	Alone	6 (13.30)	8 (12.10)	8 (9.10)
	Partner and/or children	32 (71.10)	51 (77.30)	73 (83.00)
	Parents	4 (8.90)	3 (4.50)	5 (5.70)
	Other adults	3 (6.70)	4 (6.10)	2 (2.30)
**Education,** n (%)				
	Primary	0 (0.00)	0 (0.00)	1 (1.10)
	Secondary	13 (28.30)	7 (10.60)	14 (15.90)
	Trade/Apprenticeship	2 (4.30)	4 (6.10)	4 (4.50)
	Diploma	7 (15.20)	14 (21.20)	14 (15.90)
	Undergraduate	12 (26.10)	23 (34.80)	29 (33.00)
	Postgraduate	12 (26.10)	18 (27.30)	26 (29.50)
**Employment,** n (%)				
	Full-time	13 (28.30)	19 (28.80)	31 (35.20)
	Part-time	9 (19.60)	17 (25.80)	18 (20.50)
	Looking for work	3 (6.50)	4 (6.10)	4 (4.50)
	Studying full-time	3 (6.50)	1 (1.50)	5 (5.70)
	Retired	9 (19.60)	17 (25.80)	22 (25.00)
	Home duties	4 (8.70)	1 (1.50)	2 (2.30)
	Other	5 (10.90)	7 (10.60)	6 (6.80)
**Occupation,** n (%)				
	Managers	4 (8.70)	11 (16.70)	7 (8.10)
	Professionals	13 (28.30)	18 (27.30)	28 (32.60)
	Other occupation	6 (13.00)	10 (15.10)	11 (12.90)
	Home duties or carer	4 (8.70)	2 (3.00)	2 (2.30)
	Self-employed	3 (6.50)	3 (4.50)	5 (5.80)
	Retired	10 (21.70)	17 (25.80)	23 (26.70)
	Looking for work	3 (6.50)	4 (6.10)	4 (4.70)
	Student	3 (6.50)	1 (1.50)	6 (7.00)
**Health professional,** n (%)				
	Yes	8 (17.40)	16 (24.20)	14 (15.90)
	No	38 (82.60)	50 (75.80)	74 (84.10)
**Household income,** n (%)				
	Less than $15,600	2 (4.50)	2 (3.20)	1 (1.20)
	$15,600-$52,000	13 (29.50)	16 (25.40)	16 (18.60)
	$52,000-$104,000	19 (43.20)	31 (49.20)	41 (47.70)
	More than $104,000	10 (22.70)	14 (22.20)	28 (32.60)

Table 2 indicates that, on average, participants downloaded and used a new app rarely or monthly, and downloaded and used a new health-related app never or rarely. Further, participants used the Internet to search for general information weekly or daily on average, while they used the Internet to search for health-related information monthly or weekly. A series of chi-square tests for independence indicated that there were no significant differences found between intervention groups (see Table 2).

**Table 2:** Mean ratings of eHealth and general app/Internet usage (rated on a five-point scale from 1=never to 5=daily).

		BA (n=46)	BHHP (n=66)	PLUS (n=88)					
		Mean (SD)	Mean (SD)	Mean (SD)	Chi-square tests (95% CIs for V)				
New app		2.82 (0.94)	2.68 (0.81)	2.76 (0.91)	*χ* ^2^_8_(n=199)=8.5, *P*=.39, *V*=.15, (0-0.19)				
New health-related app		1.82 (0.94)	1.88 (0.73)	1.75 (0.68)	*χ* ^2^_6_(n=199)=4.0, *P*=.67, *V*=.10, (0-0.15)				
General Web search		4.54 (3.07)	4.73 (0.54)	4.72 (0.61)	*χ* ^2^_8_(n=200)=9.2, *P*=.33, *V*=.15, (0-0.20)				
Health-related Web search		3.07 (0.90)	3.36 (0.87)	3.18 (0.96)	*χ* ^2^_8_(n=199)=7.5, *P*=.49, *V*=.14, (0-0.18)				

### Use of eHealth Tools

The majority of participants had not previously heard of or used BrainyApp or Alzheimer’s Australia’s Your Brain Matters program, and this did not differ between groups (see Table 3). Very few BA participants used an iPod Touch to access the intervention, with the vast majority using either an iPhone or iPad. BHHP and PLUS participants primarily used a desktop computer or laptop, so there was a significant association between intervention group and mode of access.

The majority of both BA and PLUS participants indicated that they were not surprised by their score on the Brain Health Survey (see Table 3; participants in the BHHP group did not have access to the survey and so were not asked this question). Across all three groups, most participants indicated that they intended to keep using the eHealth tool.

As indicated in Table 3, there were significant associations between intervention group and frequency and duration of eHealth tool use. Most BA participants reported using the app a few days a week, while BHHP participants tended to use the website weekly, and PLUS participants tended to use the program fortnightly. BA participants reported using their eHealth tool mostly for 5 to 10 minutes at a time, while most BHHP participants used their eHealth tool for 15 to 30 minutes at a time, and PLUS participants reported using their eHealth tool mostly for 5 to 20 minutes at a time. Few participants reported that they were ever unable to access the eHealth tool (eg, due to a crash).

User tracking indicated that BA participants used the app for an average of 20.5 sessions (SD 17.3, range 1-62). The average duration per session was 5.2 minutes (SD 3.5, range 0.5-13.6). BHHP participants used the website an average of 3.0 times (SD 2.4, range 1-12), for an average duration of 22.2 minutes (SD 27.2, range 0.5-137.2) per session. Finally, PLUS participants used the program an average of 2.3 times (SD 1.4, range 1-7), for an average duration of 16.6 minutes (SD 14.7, range 1.9-83.6) per session. Comparison of the self-reported frequency and duration of use with the user tracking indicated that, while broadly consistent, there was a tendency for participants to over-report their use of the eHealth tools.

**Table 3:** Use of eHealth tools.

		BA^a^ (n=46)	BHHP (n=66)	PLUS (n=88)	
		n (%)	n (%)	n (%)	Chi-square tests (95% CIs for V)
**Heard of BA/YBM** ^b^					*χ* ^2^_2_(n=196)=0.9, *P*=.63, *V*=.07, (0-0.18)
	Yes	10 (21.7)	11 (16.7)	21 (23.9)	
	No	35 (76.1)	52 (78.8)	67 (76.1)	
**Used BA/YBM**					*χ* ^2^_2_(n=195)=3.0, *P*=.22, *V*=.12, (0-0.25)
	Yes	8 (17.4)	8 (12.1)	7 (8.0)	
	No	36 (78.3)	55 (83.3)	81 (92.0)	
**Mode of access**					*χ* ^2^_8_(n=196)=113.5, *P*<.001, *V*=.54, (0.42-0.62)
	Mobile phone	23 (50.0)	3 (4.5)	2 (2.3)	
	Tablet device	20 (43.5)	13 (19.7)	11 (12.5)	
	Desktop computer or laptop	0 (0.0)	37 (56.1)	61 (69.3)	
	iPod Touch	2 (4.3)	0 (0.0)	0 (0.0)	
	Multiple devices	0 (0.0)	11 (16.7)	13 (14.8)	
**Surprised by score**					*χ* ^2^_2_(n=128)=0.4, *P*=.82, *V*=.06, (0-0.18)
	Yes, higher than expected	9 (19.6)	N/A	13 (14.8)	
	Yes, lower than expected	8 (17.4)	N/A	16 (18.2)	
	No	28 (60.9)	N/A	54 (61.4)	
**Intend to keep using**					*χ* ^2^_2_(n=190)=3.7, *P*=.16, *V*=.14, (0-0.27)
	Yes	30 (65.2)	51 (77.3)	56 (63.6)	
	No	15 (32.6)	12 (18.2)	26 (29.5)	
**Ever unable to access**					*χ* ^2^_2_(n=194)=0.8, *P*=.66, *V*=.07, (0-0.18)
	Yes	7 (15.2)	9 (13.6)	9 (10.2)	
	No	38 (82.6)	54 (81.8)	77 (87.5)	
**Self-reported frequency of use**					*χ* ^2^_12_(n=195)=40.6, *P*<.001, *V*=.32, (0.16-0.38)
	Everyday	2 (4.3)	0 (0.0)	1 (1.1)	
	Most days	12 (26.1)	4 (6.1)	5 (5.7)	
	A few times a week	15 (32.6)	14 (21.2)	9 (10.2)	
	Weekly	8 (17.4)	15 (22.7)	22 (25.0)	
	Fortnightly	4 (8.7)	13 (19.7)	27 (30.7)	
	Monthly	2 (4.3)	13 (19.7)	16 (18.2)	
	Not at all	1 (2.3)	4 (6.1)	8 (9.1)	
**Self-reported duration of use**					*χ* ^2^_8_(n=191)=26.0, *P*=.001, *V*=.26, (0.11-0.33)
	5 minutes or less	4 (8.7)	5 (7.6)	4 (4.5)	
	5 to 10 minutes	22 (47.8)	8 (12.1)	30 (34.1)	
	15 to 20 minutes	12 (26.1)	21 (31.8)	29 (33.0)	
	25 to 30 minutes	3 (6.5)	20 (30.3)	11 (12.5)	
	More than 30 minutes	3 (6.5)	9 (13.6)	10 (11.4)	

^a^BA=BrainyApp

^b^YBM=Alzheimer’s Australia’s Your Brain Matters program

### Evaluation of Intervention

The majority of participants from all three groups reported a generally positive overall impression of the eHealth tools. They also reported that the information provided was interesting, easy to understand, and easy to navigate. Again, the majority reported that the information provided was helpful, and that they learned a substantial amount from the eHealth tool.

A series of one-way between-groups ANOVAs were conducted to explore differences between intervention groups on each aspect of the evaluation. Table 4 details participants’ average responses to each of the evaluation items, and results of each of the ANOVAs.

There were statistically significant differences between groups on the variables concerning participants’ overall impression of the intervention, how interesting the eHealth tool was, how easy it was to navigate, how helpful participants found the information provided, and the amount learned, but not how easy the information was to understand. Post-hoc comparisons using the Tukey HSD test indicated that the mean rating for the BA group was significantly lower than the BHHP group’s rating on the variables concerning participants’ overall impression (*P*=.02), how interesting the information was (*P*=.03), how helpful participants found the information provided (*P*=.02), and the amount learned (*P*=.02); the PLUS group did not differ significantly from the other two groups on these variables. For the variable concerning how easy the eHealth tool was to navigate, post-hoc comparisons indicated that the mean rating for the BHHP group was significantly higher than the ratings of both the BA group (*P*=.03) and the PLUS group (*P*=.04); however, the mean score for the BA group did not significantly differ from the PLUS group.

**Table 4:** Mean ratings (rated on a scale from 1 to 5) for participants’ evaluations of the interventions.

Participants’ Evaluations	BA (n=46)	BHHP (n=66)	PLUS (n=88)					
	Mean (SD)	Mean (SD)	Mean (SD)	*F*	Degrees of Freedom	*P*	eta^2^	90% CIs for eta^2^
Overall impression	3.56 (0.84)	4.03 (0.87)	3.75 (0.89)	4.12	2,191	.02^a^	.04	(0.004-0.09)
Interesting information	3.89 (0.86)	4.31 (0.80)	4.06 (0.82)	3.53	2,189	.03 ^a^	.04	(0.002-0.08)
Easy to understand	3.52 (0.82)	3.40 (0.96)	3.44 (0.83)	0.27	2,188	.76	.003	(0-0.02)
Ease of navigation	3.64 (1.11)	4.17 (0.96)	3.74 (1.11)	4.20	2,190	.02 ^a^	.04	(0.004-0.09)
Helpful information	3.87 (0.76)	4.33 (0.90)	4.02 (0.91)	4.15	2,192	.02 ^a^	.04	(0.004-0.09)
Amount learned	3.29 (0.76)	3.79 (1.02)	3.53 (0.92)	3.86	2,188	.02 ^a^	.04	(0.003-0.09)

^a^ *P*<.05

## Discussion

### Principal Findings

In order to address the growing number of people affected by dementia, increased efforts to provide a preventative health strategy are essential [5-7,27-29]. This study aimed to explore participant engagement in targeted dementia risk reduction eHealth interventions, and to determine whether interactive eHealth tools might be more effective at engaging middle-aged members of the public than a static information-only environment. Results indicated that the majority of participants reported a generally positive experience with the eHealth tools and intended to continue using them following the intervention. However, compared to participants who used the mobile phone app (BA group), participants using the information-based website (BHHP group) reported a more positive evaluation across a range of domains.

### Use of eHealth Tools

User exposure, in terms of visiting, using, and revisiting, is an important component of examining the impact of eHealth interventions [20]. Self-reported usage of the eHealth tools indicated that BA participants were more likely to use the eHealth tool regularly, for shorter periods of time, as expected for an app (able to be used anywhere) compared to a Web-based tool (primarily available when in front of a computer). Alternately, BHHP and PLUS participants were more likely to use the eHealth tool less frequently but for longer periods of time. These differences in frequency and duration of use between the app and Web-based tools were expected due to the inherent differences in the way the two modalities are used. User tracking confirmed this pattern of frequency and duration of use, while also highlighting the tendency for participants to over-report their use of the eHealth tools.

Most participants indicated that they intended to keep using the eHealth tool following the intervention. These findings suggest that there is community interest in understanding what can be done to reduce dementia risk, and that the resources were perceived to be useful even beyond the scope of the study. It has been proposed that the primary difficulty for eHealth interventions is to engage the community for long enough so that they obtain exposure to at least the most important aspects of the program [30]. Further, previous research has indicated that continued use of eHealth programs over time is more likely to occur when earlier visits result in positive feelings [20]. Results from the present study revealed that most participants used the eHealth tool for long enough to process the information provided, and were engaged enough to use the tools on multiple occasions, highlighting the potential public health impact of these interventions. However, it is important to note that results are only available for the participants who completed the evaluation. There were quite high rates of drop-out in the present study, and there is no data available for those participants who did not complete the follow-up evaluation. It may be the case that these participants had very different experiences in terms of visiting, using, and revisiting the eHealth tools. Non-completers were more likely to be in full time employment, while completers were more likely to be retired, so time constraints may have contributed to drop-out.

### Mode of Delivery

Previous research has established that overall usability and easily accessible information are important aspects of a successful eHealth intervention [31]. In the present study, participants reported an overall positive impression of the three eHealth tools. Each of the eHealth tools were generally reported to be interesting, easy to understand, easy to navigate, as well as providing helpful information, and enabling participants to learn a substantial amount about the topic of dementia risk reduction.

All three groups had similar ratings on ease of understanding the information provided, with mean ratings falling between “just right” and “somewhat simplistic”. Further, the only significant difference between the BHHP and the PLUS groups was that participants rated the information-based website significantly easier to navigate than the interactive website. Thus, while it was hypothesized that PLUS participants would provide a more positive evaluation of the interactive tool compared to those who accessed the static information-only website, results indicated that, overall, the two versions of the website were rated equivalently. This is at odds with prior research which has indicated a strong user desire for interactive components of an eHealth intervention [25,31,32] and may indicate shortcomings with the design of the interactive website tools for those participating in this study.

There were, however, a number of significant differences between the BA and BHHP groups, with the BHHP group rating the information-based website more interesting, helpful, and favorable overall, reporting that they learned more, and finding their eHealth tool easier to navigate. While the popularity of using mobile phone apps to deliver health information is growing rapidly [33,34], as yet there is limited evidence as to their effectiveness [35] and the current findings suggest that traditional modes of eHealth delivery may be more appropriate for middle-aged populations. Specifically, as most participants were in their fifties, and downloaded and used a new app rarely, the limitations of BrainyApp may have had more to do with the mode of delivery than the app itself; as participants appeared to have limited experience using apps. In addition, there was no specific instruction provided on how to use the app, or how to make the most of the interactive components (such as the survey, apart from some basic instructions included in the app itself). As a result, participants may have experienced some confusion around how to access the relevant information.

### Strengths and Limitations

The evaluation of publicly available dementia risk reduction resources represents a major strength of the present study, as it promotes a greater understanding of the features that contribute to user engagement with these resources. The results of this study have the potential to inform future developments in dementia prevention initiatives for the Australian and international communities.

However, there were a number of methodological limitations to the present study. Firstly, the results may not be generalizable to the Australian population as a whole, as the sample consisted predominantly of older, female, highly educated participants. There were also large drop-out rates, particularly for the BA group. Additionally, there were significant differences in education level and employment status between those who completed the follow-up, and those who dropped out, which may also limit the generalizability of findings. However, participants were randomly allocated into groups in an effort to limit the impact of these issues. Previous research has indicated that people who are highly motivated to live a healthy lifestyle are more likely to use the Internet for health-related information, and that older, highly educated people are more likely to revisit these information sources over time [20,30]. This highlights the importance of targeting interventions such as the ones evaluated here for a broad range of demographic groups. More work is needed to determine how to attract less motivated, younger, and less educated people to use online dementia risk reduction resources.

A further limitation is that apps are designed to be used differently and can offer very different features compared to Web-based tools. As a result, the user experience may not be directly comparable between these two modalities. All groups engaged with their eHealth tool as expected, such that frequency and duration of use differed between modalities. BA participants used the app more frequently but for shorter durations, compared to BHHP and PLUS participants less frequent but longer duration use of the Web-based tools. However, the app and interactive website were designed to provide similar information and resources within the parameters of each modality, while the information-based website was used to compare interactive and static tools. The findings of this study provide important information about user preferences within and across the different modes of delivery of dementia risk reduction information.

There were also a number of technical issues across the eHealth interventions. The user tracking was limited in that it was unable to record BA participants’ final session, and was unable to record the duration of the last page visited for BHHP and PLUS participants if they did not log out of the session; thus, the user tracking information is somewhat incomplete. Further, the large drop-out rate for BA participants (following randomization to that group) may have been due in part to the technical requirements for installing the app on their device (participants had to sign up to a third-party app in order to install the research version of BrainyApp, rather than downloading it from the App store). While necessary to facilitate user tracking, the process proved difficult for some participants and the majority of drop-outs happened at this stage. Some participants reported difficulties with the lack of guidance provided on how to use the app. While this is an inherent feature of apps which are meant to be intuitive, the unfamiliarity with using apps of the demographic group involved in this study may have contributed to the higher drop-out rate in the BA group.

### Implications and Future Research

Previous research identified that, while an information-based dementia risk reduction website was reported to be useful and relevant, users wanted more interactive and personalized resources [25]. Research into other eHealth interventions has also reported a user desire for interactivity (eg, physical activity interventions [31]). However, the results of the present study indicate that the information-based website received a more positive evaluation than the two interactive learning environments. Thus, it may be the case that information-based resources are more appropriate for some groups of people than interactive resources. For example, participants in the present study likely volunteered because they had an existing interest in brain health, and thus may have been seeking more detailed information than an app can provide. Interactive resources may be more beneficial to people who have little prior interest or knowledge about brain health and dementia risk reduction, as a means to engage them with the topic.

Nevertheless, the results of the present study provide an important platform from which to improve public health dementia risk reduction resources. Further research is required to determine whether there are specific interactive components that can be used to improve and enhance the way information is provided to the general community, above those gained by providing static information alone. Future research should also determine whether resources such as the ones evaluated here have the potential to improve dementia risk reduction knowledge and motivation, and to change people’s behaviors toward a more brain healthy lifestyle.

### Conclusions

The results of the present study demonstrated that, overall, participants from each of the three intervention groups reported a generally positive experience with the targeted dementia risk reduction eHealth tools. In particular, participants who used the information-based website reported a more positive evaluation, across a range of domains, than participants who used the mobile phone app. These findings will inform future developments of Alzheimer’s Australia’s dementia risk reduction resources.

## Abbreviations

AUD: Australian dollar
BA: BrainyApp (mobile phone/tablet application) group
BHHP: Brain-Heart Health Program (information-based website) group
PLUS: Brain-Heart Health Plus Program (interactive website) group
